# Would you offer contraception to a 14-year-old girl? Perspectives of health students and professionals in Dar es Salaam, Tanzania

**DOI:** 10.1186/s12978-021-01294-6

**Published:** 2021-12-11

**Authors:** Dorkasi L. Mwakawanga, Ever Mkonyi, Stella E. Mushy, Maria Trent, Zobeida Bonilla, Agnes F. Massae, Gift G. Lukumay, Lucy R. Mgopa, Inari Mohammed, James Wadley, Michael W. Ross, Sebalda Leshabari, B. R. Simon Rosser

**Affiliations:** 1 School of Nursing, Muhimbili University of Health and Allied Sciences (MUHAS), Dar es Salaam, Tanzania; 2 Division of Epidemiology and Community Health, School of Public Health, University of Minnesota, 1300 S. 2nd St. #300, Minneapolis, MN 55454 USA; 3 Schools of Medicine and Public Health, Johns Hopkins University, Baltimore, MD USA; 4 School of Adult and Continuing Education, Lincoln University, Philadelphia, PA USA; 5 Department of Family Medicine and Community Health, University of Minnesota, Minneapolis, MN USA

**Keywords:** Adolescent, Family planning, Provision, Health professionals, Health students, Tanzania

## Abstract

**Background:**

Rates of unplanned adolescent pregnancy and unsafe induced abortions are very high in Sub-Saharan African countries including Tanzania. Despite their availability and accessibility, modern family planning methods are reported to be critically underutilized by adolescents. This study is part of a broader study that aims to develop a curriculum that will be used in training health professionals by investigating the sexual health training needs of health providers and students in Tanzania.

**Aim:**

This study describes the perceptions of health professionals and students on the provision of contraceptives to adolescents.

**Methods:**

Qualitative formative assessment type of research was conducted using 18 focus groups stratified among health professionals and students (midwives, nurses, and medical doctors). Study participants were presented with the theoretical scenario of a 14-year-old girl who sought contraceptive services at a family planning clinic. This theoretical scenario was used to determine how health professionals and students would handle the case. Thematic analysis guided the examination and determination of data results.

**Results:**

Three main themes emerged from the data, including (1) knowledge about the provision of contraceptives to adolescents, (2) perception of the adolescents’ right to contraceptive use, and (3) barriers to the provision of contraceptives to adolescents. Participants stated that having a baseline knowledge of contraceptive services for adolescents and their rights to contraceptives would trigger their decision on offering the contraceptive. On the other hand, being unaware of the reproductive health rights for adolescents, judgmental behavior of providers, as well as religious and cultural dynamics were all found to be major barriers for providers to offer contraceptive services to the 14-year-old adolescent girl in the theoretical scenario.

**Conclusion:**

These findings support the need for comprehensive sexual health education in Tanzanian health professional training programs.

## Plain language summary

In Tanzania, adolescent pregnancies are culturally unwanted, abortion remains illegal and yet the utilization of family planning services among adolescents is still low. The community myths and misconceptions about the side effects of contraceptives, healthcare providers’ attitudes, and barriers within the healthcare system hinder adolescents’ utilization of family planning services. The effective use of family planning methods will prevent the use of unsafe induced abortions and hence reduce morbidity and mortality among young women. This will be possible only if healthcare providers are equipped with the knowledge and skills to address these sexual and reproductive health challenges among adolescents in Tanzania. Therefore, in this study, we decided to explore the perceptions of health professionals and students on the provision of contraception to adolescents. We conducted 18 focus groups stratified among health professionals and students (midwives, nurses, medical doctors). Participants were supplied with a theoretical scenario wherein a 14-year-old girl presented to a family planning clinic seeking contraceptive services to determine how health professionals and students would handle the case. We found that the decision of providers with regards to offering contraception is highly dependent on having a baseline knowledge of the rights and contraceptive services available to adolescents. Some barriers identified as reasons for providers not to offer contraceptive services include a lack of awareness of the reproductive health rights for adolescents, judgmental behavior (implicit and explicit biases among providers), as well as religious and cultural dynamics. Participants also acknowledged that the presence of guidelines and proper training among providers might avoid the infringement of adolescents’ rights to sexual and reproductive health services. In conclusion, these findings support the need for comprehensive sexual health education in Tanzanian health professional training programs.

## Background

Tanzania is a country with significant sexual and reproductive health challenges, yet health professionals and health students receive almost no training on how to address sexual health concerns involving the implementation of family planning services in adolescent groups [1]. Sub-Saharan Africa has the world’s highest rates of adolescent pregnancy leading to poor pregnancy outcomes [2]. Unsafe induced abortion contributes notably to the morbidity and mortality rates observed among young women [3]. East Africa had an estimated 2 million unsafe abortions in 2008, with 3.6% of those in women aged 15–44 [4], and accounting for 16–25% of maternal deaths [3]. In Tanzania, the number of childbearing adolescents aged 15–19 years has gradually increased from 23% in 2010 to 27% in 2016, and the adolescent fertility rate has increased from 116 to 132 per 1000 girls during that same period. By the age of 19 years, almost 56.7% of adolescents have had their first child [5]. Most women seeking abortions are young, single, and anxious [6], which in the Tanzanian cultural context suggests that many adolescent pregnancies may be unwanted. Abortion in Tanzania is illegal but widely practiced; however, given legal restrictions, it is difficult to obtain reliable information to assess the magnitude and safety of abortive procedures [7]. The main strategy to prevent the use of unsafe induced abortions is the effective use of modern family planning methods. Despite efforts put in by the Tanzanian government to ensure accessibility of family planning to every woman of reproductive age, significant delivery gaps for providing quality family planning services to adolescents remain. Most married (85%) of the married and unmarried (60%) adolescents do not use family planning services [5].

Compared to adults, pregnant adolescents also face disproportionately higher levels of risk for multiple adverse health outcomes such as sexually transmitted infections (STIs). Additionally, they face heightened discrimination as well as impaired socioeconomic status when compared with adult women. Common discriminatory events include compulsory expulsion from school, higher rates of unemployment and being expelled from their home. About half of pregnant adolescents in Tanzania have at least one STI, with the most commonly diagnosed STI being herpes simplex virus (HSV-2) [8]. Furthermore, 27% of pregnant girls in Dar es Salaam, the largest Tanzanian metropolitan area, had severe wasting (severe acute malnutrition), and 48% of their babies had a low birth weight [9].

Several structural factors, including common cultural myths and misconceptions about the side effects of contraceptives, healthcare providers’ attitudes, and the barriers within the healthcare system hinder adolescents’ utilization of family planning services [10–12]. Healthcare professionals themselves may be perceived as barriers when they impose their personal values or beliefs on their adolescent patients [10]. To improve adolescent access to contraceptives and reproductive health outcomes, health professional training programs must incorporate adolescent development and sexual healthcare in the standard curricula. Midwives, nurses, and physicians provide the majority of healthcare in Tanzania. As such, they are respected community health experts who provide public health advice to local, regional, and national leaders who shape public health policy. The perspectives, practice, and advocacy efforts of these trusted health professionals also impact community norms. Yet, until recently, they received no formal training in sexual healthcare; particularly, there was no direct sexual health education or skills training for primary care delivery.

For these reasons, we affirm that providing sexual health education to midwifery, nursing, and medical students is a critical issue in Tanzania. At the Muhimbili University of Health and Allied Sciences (MUHAS) in Dar es Salaam, we adapted a PAHO/WHO sexual health curriculum for healthcare providers and piloted it with midwifery, nursing, and medical students. The pilot demonstrated the feasibility, acceptability, and promise of the curricular intervention for improving health workers' knowledge, comfort, and skill level in providing sexual healthcare to patients [13]. In this paper, we describe the results of formative research conducted in 2019 to explore the perspectives of health professionals and university students in health professional training programs on the provision of contraceptive family planning methods in a theoretical scenario involving a 14-year-old girl as a sexual and reproductive health problem. This research aims to develop an Afro-centric sexual health curriculum for midwifery, nursing, and medical students.

## Methods

### Research design

This research employed a cross-sectional qualitative study to explore the effectiveness of Afro-centric sexual health training for students in the health professions in a large university in Tanzania. The study design was considered appropriate to inform the development of an Afro-centric, culturally specific curriculum for the training of midwifery, nursing, and medical professionals in sexual health. This study was conducted by a collaborative team from Muhimbili University of Health and Allied Sciences in Tanzania and the University of Minnesota in the United States. The team focused on the interdisciplinary training of health professionals.

In 2019, 18 focus groups were examined. The exploratory qualitative design was stratified to examine findings across the 3 main healthcare disciplines by conducting 3 focus groups among each stratum (i.e., midwifery, nursing, and medicine) and 9 focus groups for each level of experience (9 among practicing clinicians and 9 among students). This design allowed the team to assess differences across the 3 main types of providers, and to capture the experiences in the practice of providers as well as the training needs of current students. The focus group method was chosen because of the strength of this method to generate group interactions in response to the key questions presented. Furthermore, focus groups proved to be an efficient method in assessing current practices and exploring different experiences while also facilitating discussion about the variations and nuances between individuals with power differentials [14, 15].

### Study setting

This study was conducted at the Muhimbili University of Health and Allied Sciences and 3 selected hospitals in Dar es Salaam, Tanzania. Muhimbili University trains the largest number of future health professionals in Tanzania. Apart from training health and allied personnel, the university has conducted key research that has been instrumental in informing Tanzanian health policy. This study included students and professionals across 3 health disciplines. Students came from 2 of the 5 schools within the university: namely, the School of Nursing and the School of Medicine. The professionals included nurses, midwives, and physicians who practice in 2 public hospitals and 1 private hospital in Dar es Salaam.

### Study participants and recruitment

A total of 121 health professionals and students participated in this study (61 health professionals and 60 students). Midwifery, nursing, and medical students who had reached at least their fourth year were recruited from the university. Professional midwives, nurses, and physicians with at least 2 years of clinical experience were recruited from the 3 major public and private health hospitals in Dar es Salaam. A purposive sampling approach [16] was used to recruit a diverse sample of participants, whereby health providers were recruited from different departments or clinical sites. When more than 1 health professional in a department or clinic met the criteria, the health provider with the most experience or expertise was enrolled for participation in the study.

Students were recruited from the main campus of the university. Fliers were posted on several notice boards 1 month before the data collection period. The recruitment of students was conducted with the assistance of the class representative of each discipline. Similarly, for providers, the head of each selected department or clinic in the hospital assisted with the process of recruitment of the targeted healthcare professionals.

### Procedures

Each focus group consisted of between 5 and 8 participants and lasted between 60 and 90 min. The groups were conducted within the university (for students) or at their hospital of employment (for providers) to ensure convenient access and privacy. The focus groups were conducted in a conference room or designated rooms suggested by the participants within a respective hospital or university premise. Only 1 focus group was conducted each day to allow time for reflections and debriefing. Each group was led by a bilingual trained moderator who asked questions in Kiswahili assisted by a co-moderator who took notes and observed. Consent forms were obtained and focus group procedures were explained before initiating data collection. General demographic information was collected at this point. Participants were informed that their participation was voluntary, they could choose not to continue at any time, that all responses would be kept confidential, and that the session was being audio recorded. Participants were instructed to write their first name (or an alias) on tent cards and were invited to respond in either Kiswahili or English. The moderator stressed that there were no right or wrong answers and encouraged members to share their experience even if they perceived it to be different from other members. Following the procedures recommended by Krueger and Casey [15] the moderator and assistant moderator met immediately after the focus group discussion for the debriefing session to develop additional notes, discuss first impressions and salient themes, and address any practical or logistical concerns related to conducting the discussion.

The focus group interview guide consisted of 14 questions that focused on the participants’ current practice (for professionals) or their most recent completed rotation (for students). The initial questions focused on participants identifying the most common sexual health challenges of patients in Tanzania. The groups were then asked to consider several clinical case studies and discuss how each case would be handled at their clinic or hospital. The clinical cases included rectal gonorrhea, erectile dysfunction, domestic violence, child sexual abuse, masturbation in adolescence, and offering contraceptives to adolescents. In response to the need to develop training curricula with content on adolescent sexual and reproductive health, this analysis focuses on the following scenario presented to participants describing a clinical encounter with a female adolescent: “a 14- year-old girl comes to your clinic asking for contraceptives. How would this be handled?”.

### Data analysis

Audio recordings were transcribed verbatim then translated into English. Deductive-inductive, team-based coding was implemented for codebook development and data analysis [17]. Thematic analysis was conducted following procedures outlined by Braun and Clarke as well as Saldana [17, 18]. To ensure reliability, the coding was done manually in pairs. Discussions were conducted about the codes, their applications, and their definitions for the purpose of refining the codes. During the coding process, unresolved discrepancies and disagreements with the codes or coding process were discussed and resolved with the team as a whole. This iterative process and peer debriefing method continued until differences were resolved and agreement was reached on the meaning of the codes, their applications, and the coding process. This peer debriefing method was used for verification, confirmation, and codebook refinement [19]. Individual codes were then organized into larger themes and sub-themes.

## Results

The overall sample consisted of about an equal number of students and experienced providers (n = 60 for students and n = 61 for providers). The sample was predominantly female (59.5%), with a mean age of 34.3 years overall; 25.5 years (SD = 3.1) for students, and 43.1 years (SD = 9.5) for the providers. Students had 4 years of experience (in school) while providers had a mean of 14.6 years (SD = 9.8) of clinical experience. The demographic characteristics of the sample are described in Table 1.

**Table 1:** Demographic characteristics of the participants

	Midwifery	Nursing	Medicine	Total
1. Sample size				
Students	20	19	22	61
Providers	21	21	18	60
	41	40	40	121
1. Gender				
Students	14 M; 6F	13 M; 6F	11 M; 11F	38 M; 23F
Providers	0 M; 21F	5 M; 16F	6 M; 12F	11 M; 49F
	14 M; 27F	18 M; 22F	17 M; 23F	49 M; 72F
2. Age (in years)				
Students: Mean	27.7	25.1	24.0	25.5
Range	23–37	23–27	22–28	23–37
Providers: Mean	44.5	41.1	43.5	43.1
Range	26–58	24–59	31–62	24–62
3. Experience (in years)				
Students	4	4	4	4
Providers: Mean	18.0	13.1	11.9	14.6
Range	4–38	2–30	4–25	2–38

N = 119 healthcare students and providers, Tanzania

**Fig. 1:**
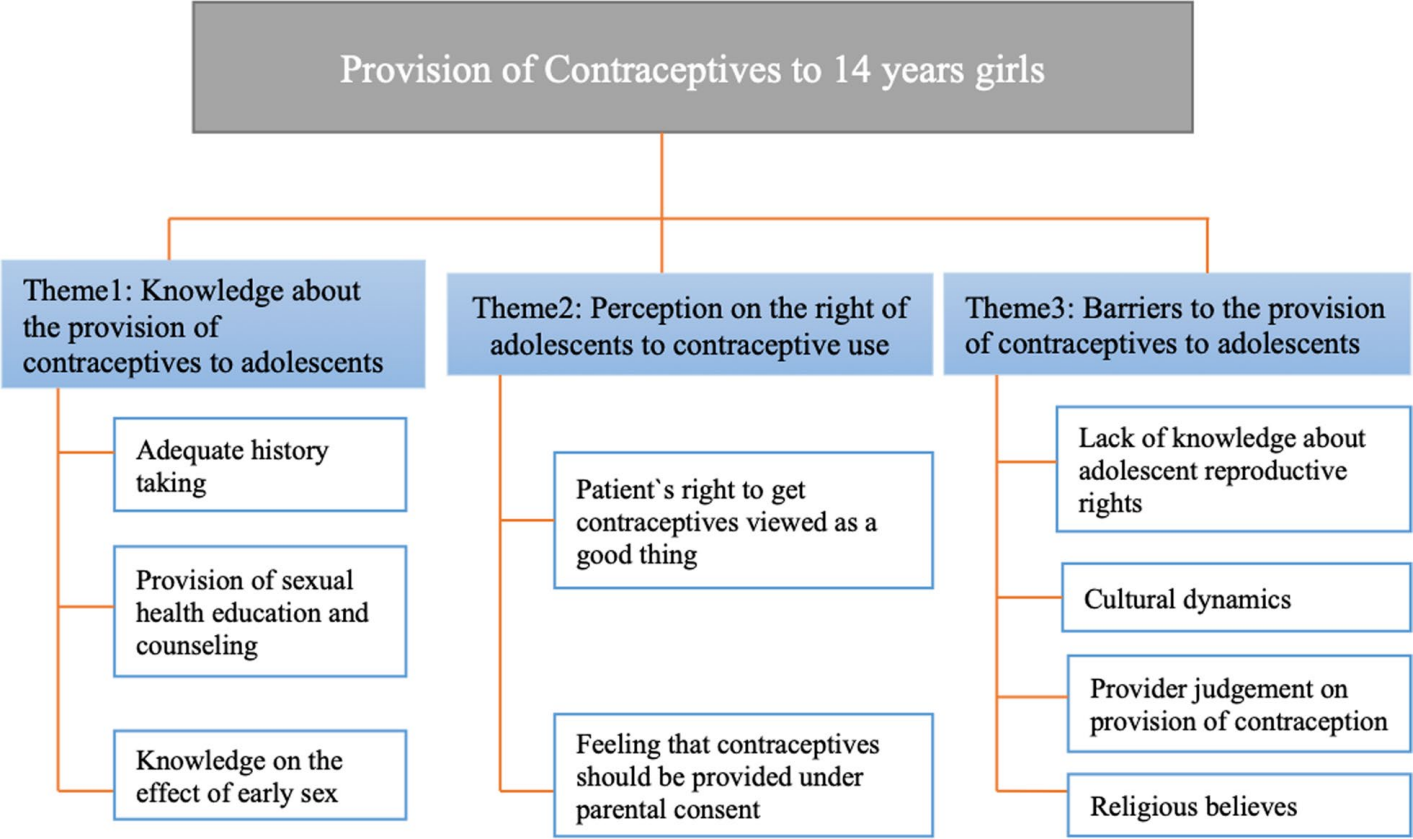
Summary of the findings

The analysis of the responses to the case study about the clinical encounter of a 14-year-old girl seeking contraceptive methods revealed three main themes: (1) knowledge about the provision of contraceptives to adolescents, (2) perception of the rights of adolescents to contraceptive use, and (3) barriers to the provision of contraceptives to adolescents. Figure 1 summarizes these key findings.

1. Knowledge about the provision of contraceptives to adolescents

When participants were asked to explain how they should handle the case of a 14-year-old girl who comes to a clinic asking for contraceptives, 3 sub-themes were generated under the main theme of knowledge from both the students’ and providers’ perspectives. These sub-themes are as follows: adequate history taking, providing sexual health education and counseling, and examining the physical and mental effects of adolescent sex on the wellbeing of the teen.

- Adequate history taking

Participants described history taking as integral to understanding the patient’s theoretical situation. This was widely discussed by both health professionals and students. Most participants considered taking a patient history as the initial step when handling a case where they are providing contraceptive methods to a client. They reported that it is very important to know the reason the patient is seeking contraceptives or should be prescribed contraceptives.

I would take a history to find out whether she has already started having sex. (…..) Also, I would find out about her family: where she is coming from, what kind of environment she has at home. Maybe she is being enticed by her friends and peer groups (…...) I would ask her questions about why she wants to use those [contraceptive] methods; maybe she had sex and she needs to take these pills to prevent pregnancy (Medical Professional FG10)

I would ask her what she knows about sexually transmitted diseases...Is she sexually active and why does she need contraceptive methods? Maybe something is going on here, we all know that a 14-year-old child may be a class 7 of primary school. Once I see her enter the office something that comes to mind is that maybe she was raped? (Medical Student FG4)

Another concern raised by participants in taking a medical history is the importance of ensuring that the patient is not being forced to have sex. They would question the patient about her sexual relationship(s), her safety in the relationship(s), her living environment, and other factors to ensure that she is not being forced to have sex and that seeking contraceptives is her choice.

If you have such a child [as a patient] you need to spend enough time counseling her because first of all, she is not married and you would like to know the kind of relationship she has, probably with an elderly man. And you may ask if she is in school, does she live with her parents? So, there are a lot of issues which we have a real need to find out. (Midwife Professional FG11)

b) Provision of sexual health education and counseling

Participants reported that it was essential to provide education to the patient in this scenario about the effects of early pregnancy and early sexual engagement. Health education described in this context included complications that may arise during labor and delivery, the increased risk of cancer due to early exposure to sexual pathogens that can be acquired during unprotected intercourse, and the importance of using dual protection (contraceptives and condoms) for pregnancy and sexually transmitted infections (STIs) including human immunodeficiency virus (HIV).

Also, I would educate her since she has come for family planning because she knows family planning protects against unplanned pregnancies and early pregnancies. I would educate her on various ways of family planning which she can use, such [as] condoms, that can protect against sexual diseases and other infections (Medical Professional FG16)

I would educate her on the effects she might experience when engaging in sexual intercourse at a premature age. She wants the methods of family planning to protect against pregnancies, but she should know that starting sexual intercourse at an early age may lead to various effects including cervical cancers (Midwife Professional FG11)

Another participant emphasized that informing the patient about the types of contraceptive methods available, how they work to prevent pregnancy, and the benefits and side effects of each method would facilitate the process of the patient making an informed decision regarding their sexual activity and their use of contraceptives. Also, one participant reported that he would first assess the theoretical girl’s baseline knowledge and respond to her questions if she has any before giving her the contraceptive(s) and family planning information.

Before offering [contraceptives] I must educate her about the use of contraceptives but also inform her of the side effects, complications and (......) knowing their benefits and risks (Nursing Professional FG12)

I would start by asking how much she knows about family planning (FP) and why she wants to use it, and then, I would help her to learn more about which contraceptive methods are available to help her, and once she has understood [and made a decision about which method is best for her], I will grant her request (Medical Student FG4)

c) Knowledge on the effects of early sex

The participants from the midwifery and medical professional focus groups discussed the negative effects of exposure to early sex and contraceptive use on cancer risks and infertility and how these may impact the future of the young patient. They debated the potential effects of exposure to early sex and contraceptive use that may increase the risk for cervical cancer and infertility due to exposure to STIs. They explained that they would inform the patient about the potential negative reproductive health problems that early sex may have in later life.

A 14-year-old girl needs contraception. First, I would thank her for coming as a girl, and then after, if it seems she has engaged herself in a sexual relationship. I would educate her that her age is still yet to have these methods, and the effects she might face. As we have seen earlier, she might face infertility since she engaged earlier in a sexual relationship (Midwife Professional FG11)

When such a girl comes before you listen to her because she is like you, then you give her the ABC’s of early sex. Also, advise her that even though she is young, if she doesn’t use condoms she will suffer from cancer (Medical Professionals FG10)

2) Perception on the right of adolescents to contraceptive use

The majority of participants had a positive perception towards women’s reproductive rights on the contraceptive use. However, the decision to provide contraceptives to a 14-year-old girl raised a lot of uncertainties. Some participants stated that they would provide the contraceptives right away because they viewed the right of the patient to receive contraceptives as a positive health decision. Others reported that they would not provide contraceptives without getting consent from the parents first.

- Patients’ right to receive contraceptives viewed positively

Most participants said that they would offer contraceptives to protect the patient from unplanned, early pregnancy and seemed to understand both the nuance in providing care and the adolescent’s rights under the law. Some parents who bring their young girls into family planning clinics prefer the healthcare provider to give contraceptives to the adolescent without their knowledge. The majority of participants stated that according to laws and regulations, girls have the right to receive contraceptives regardless of their age. Moreover, participants (especially midwives and nurses) showed concern about giving girls a high-priority service (contraceptives) while also maintaining the requisite degree of privacy as an essential factor in providing the best patient care.

For me, I would offer the methods. I would inform her about all the methods for her to choose from and I wouldn’t involve parents because some parents will deny the child from getting what she wants (contraceptives). But for the sake of preventing an unplanned pregnancy or an early pregnancy, I would offer contraceptives to her. (Medical Student FG5)

As far as the law is concerned regarding contraception, she has the right to get contraception if she wants. But we have to educate her on the available types of contraception and advise her not to get pregnant because of her age because if she conceives, she will be exposed to more dangerous issues (Medical Professional FG10)

She will be given a priority because of her age, because when she meets adult women, they will start thinking badly about her and so when, as a provider, you identify her age you have to call her and give her priority in service, give her proper counseling like other women, [so that] she will be able to choose and get the appropriate services as needed (Midwife Professional FG17)

Despite these factors, the majority of participants stated that they would offer contraception to the patient. A minority of participants were against providing contraceptives and asserted that they would rather advise the patient to abstain from sexual intercourse and concentrate on her school studies.

“I must take a history to find out whether she has started having sex. If you find that she has not started sexual intercourse it is best to advise her to concentrate on her studies. I will discuss with her and see whether she can abstain because of her age as she is still young (…...) (Medical Professional FG10)

b) Feeling that contraceptive should be provided under parental consent

Some participants stated that because the girl in this scenario is only 14 years old, she is still underage and cannot provide informed consent for healthcare services. They stated that they would ask her to bring her parents, preferably a mother, to give consent for her to access family planning services like contraceptives.

A 14-year-old girl cannot give consent for herself. Therefore, for her to get contraception, her parents have to consent for her. So, I will ask her to go and bring her parents. After she brings her parents, I will educate her and her parents about contraception, for her to make a choice. And then I will offer her the method of her choice after consent from the parent (Midwife Student FG9)

3) Barriers to the provision of contraceptives to adolescents

The provision of quality care among adolescents is hindered by a lack of knowledge on adolescents' reproductive health rights/policy, cultural dynamics, judgmental provider behavior, and religious beliefs.

- Lack of knowledge about adolescent reproductive rights

Most participants had inadequate knowledge on what formal reproductive health policy/guidelines say about the provision of contraceptives to adolescents. The absence of this knowledge places participants and providers in an unnecessary dilemma during clinical service visits and serves as a barrier to care.

They are abandoned by the law because they are not adults. I would counsel her to abstain from having sex but in the back of my mind, I would know that she is not going to stop…….it is an ethical dilemma (Medical Professional FG13)

Other participants appeared to have put themselves in the position of conducting a parenting role instead of providing care as a health professional.

“Even me I would not provide contraceptives, though giving her is okay. I don’t know what to do because she is still young, I would ask her why she would engage in those issues. Later I would request her to bring her boyfriend to give them counseling...! (Midwife Professional FG11)”

Students appeared unsure about the patient’s rights under Tanzania law and asked other group members whether the policy allows a 14-year-old girl to consent to receive family planning services. One participant said:

Maybe let’s talk about our policies in Tanzania, regarding the age of the child to consent for issues regarding health. Is a 14-year-old girl allowed to give consent? (Medical Student FG4)

Another participant stated that the law does not allow adolescents to use contraceptives but then proclaimed that it has to change. The law seemed to be a setback for the decision to offer contraceptives to the patient in the presented scenario.

In case a 14-year-old girl comes, these laws do not allow her to be given family planning. This is a setback and most are getting unplanned pregnancies as a result of this. Therefore, some laws should be changed to allow family planning to be given to 10-year-old girls and above (Nursing Student FG1)

b) Cultural dynamics

The scenario that a 14-year-old girl was having sex and asking for contraceptives was viewed by some as a violation of African culture. This also affected the decision of most participants to provide contraceptives. The majority who stated that they would not give contraceptives said that it was because they wanted the girl to focus on her studies rather than being engaged in sexual activities. They expressed concern that this behavior (sex) would influence the development of other dangerous habits/behaviors.

The fact is that in Europe such things are normal but in African traditions, this has a little challenge. It’s something that helps but based on African culture, young children will get involved in dangerous behaviors in case they are provided contraception. Therefore, I would try to create an environment for her to concentrate on her studies, and honestly, I wouldn’t give her the family planning pills (Midwife professional FG11).

c) Provider judgment on the provision of contraception

The judgmental behavior and biases of providers were seen to be a barrier to the provision of contraception to a 14-year-old girl. Participants reported several reasons as to why they or others may not be offering the contraceptives to the patient. This included reasons such as: her age (under 20, too young to be having sex), her studies (supposed to be at school), and shame (she should be ashamed of getting involved in sexual activities at a very young age).

I think the thing is, you may let them go and take the pills but they have to go to the Reproductive and Child Health clinic for it and once they meet with these grandmother nurses, they will be told as you have already started having sex…. are you not ashamed? (Medical Professional FG13)

Therefore, she should be counseled to abstain until she reaches at least 20 years old, and when she has completed school/studies (Midwife Professional FG11)

d) Religious beliefs

Some participants worried about having sex prior to marriage, expressing that it may be perceived as a sin. Thus, they said they would not provide contraception to a 14-year-old girl. Instead, participants reported that they would advise the patient not to have sex until she accomplishes her studies and gets married.

She is still young to me. On the side of religion to have premarital sex is a sin and the girl is still young. Let her wait and finish school then get married like what my colleagues said (Nursing Professional FG18)

## Discussion

The key finding in this study is the lack of overarching standards in practice. While most health professionals stated that they would provide contraceptives to the 14-year-old girl in this scenario, not all agreed. Some participants appeared to be unaware or uncertain of what the reproductive health policy in Tanzania states about adolescents using contraceptives. While several providers acknowledged that the girl had the right to receive contraceptives, their judgmental behavior as well as the cultural and religious beliefs of some participants were found to be the main barriers to the provision of contraceptives to adolescents.

Across the focus groups, the provision of contraceptives to adolescents was perceived negatively and as an encroachment on African culture among health professionals in addition to those in training. These findings may explain at least in part why the Tanzanian National Demographic Health Survey reports that 85% of unmarried women do not use modern family planning methods [5]. A study from Nigeria and Kenya among providers on contraceptive counseling and care of female adolescents reported similar findings in that the majority of providers did not provide contraceptives for unmarried adolescents and discouraged them from using contraceptives because they believed that to do so would be promoting sexual promiscuity [20, 21]*.* This differs from practices in European Union countries where provider perspectives on adolescent family planning are generally positive, adolescent-friendly sexual and reproductive health services and care facilities are easily accessible, and contraception use among adolescents is high [22]. However, some African countries have not developed formal policies or recommendations that guarantee respect, confidentiality, and the possibility of consulting a physician confidentially, without parental or guardian knowledge [22]. Differential practices observed between African and Western countries may be due to the differences in health professional training, resources, and cultural and traditional taboos.

Although some of the participants from this study were willing to offer contraceptives to the patient in the scenario presented to them, many lacked the requisite knowledge on the reproductive health rights and policies regarding adolescents and the use of modern family planning methods, and the importance of non-judgmental communication skills to deliver care to adolescent girls. These gaps in knowledge may be reflective of deficiencies in the content of the existing health professional training curricula and a need to develop a culturally relevant curriculum for health professional students and practicing providers focused on sexual health issues, including adolescents’ right to family planning and other sexual health services. Introducing a new module in the nursing, midwifery, and medical school curriculums could be an avenue for improving the quality of care and strengthening support services for adolescents [11, 23]*.* Integrating components to address knowledge deficits regarding adolescent reproductive health rights and Tanzanian policies for the delivery of quality family planning services are essential for adequate adolescent care delivery and a reduction in health disparities.

This research also suggests that it will be important for a new curriculum to address how the cultural dynamics, religious beliefs, and judgmental perspectives and behaviors of providers influence the quality of health services and contribute to health disparities in Tanzania. These factors were found to be core impediments to service delivery for adolescents when there is a need to increase the number of adolescents seeking care and family planning services. It is important that adolescent patients feel safe and have private and confidential discussions with trustworthy health professionals to facilitate disclosure and limit the possibility of stigmatization [20, 24, 25]*.* Healthcare professionals in the Tanzanian context appear to diverge from their role as healthcare providers with some adopting a parental role instead. This finding aligns with other studies where the judgmental attitudes of health service providers intermingled with cultural, religious, and traditional values which then influenced provider practice and the standard of care [26, 27]*.* Addressing knowledge, attitudes, behavior, and effective communication strategies that consider adolescent development can improve provider performance. Directing more attention to these aspects of care may contribute to improving access to high-quality reproductive health services, including contraceptive prescriptions. The availability of information on adolescent sexual and reproductive clinical guidelines, public health policies, and laws should optimize the rights of adolescents to access family planning services. The presence of these guidelines and proper training among providers may help to minimize the deprivation of adolescents’ rights to sexual and reproductive health services [23, 28–30].

### Strengths and limitations

This study must be considered in light of several strengths and limitations. This study’s strength and credibility are enhanced by triangulating data sources from focus groups with a large number of healthcare providers from private and public hospitals as well as health professional students. Though health professional instruction is executed in the English language, the use of bilingual moderators and integration of the Kiswahili language enhanced the quality, depth, and accuracy of data. Kiswahili is the national language of Tanzania and is spoken comfortably by all participants. The use of Kiswahili during data collection enabled facilitators to ensure that concepts and cases were well understood, limited the risk of misinterpretation, and reduced the risk of distortion of participants’ accounts due to translation during the analysis process [31]. Even so, data were only collected from health professionals living in Dar es Salaam, so generalizability outside of Dar es Salaam to other urban or rural Tanzanian contexts is limited. Furthermore, while the moderators encouraged all participants to share and encouraged discussion of differing views, some participants may have over- or under-reported their experiences in the context of the group experience [32].

## Conclusions

Healthcare students’ and professionals’ perspectives on offering contraception to a 14-year-old girl was affected by a lack of knowledge about sexual and reproductive health rights for adolescents. Moreover, the personal, cultural, and religious lens through which they viewed the case affected their decision on providing contraception. This calls for the provision of on-the-job training for healthcare professionals about the rights of adolescents to procure contraceptives so that providers can direct adolescents to age-appropriate reproductive and sexual health services. Additionally, this work offers important data as a basis for the revision of the content in the previously used curriculum for health professionals to improve access and experiences for adolescents in the healthcare system and reduce health disparities among adolescent girls and young women in Tanzania.

## Data Availability

The datasets used and analyzed during the current study are available from the corresponding author on reasonable request.

## Abbreviations

MUHAS: Muhimbili University of Health and Allied Sciences
NICHD: National Institute of Child Health and Human Development
NIMR: National Institute of Medical Research
IRB: Institutional Review Board
STIs: Sexually transmitted infections
PAHO/WHO: Pan American Health Organization/World Health Organization
HIV: Human immunodeficiency virus
FP: Family planning
